# Symmetric Kullback-Leibler Metric Based Tracking Behaviors for Bioinspired Robotic Eyes

**DOI:** 10.1155/2015/714572

**Published:** 2015-11-11

**Authors:** Hengli Liu, Jun Luo, Peng Wu, Shaorong Xie, Hengyu Li

**Affiliations:** School of Mechatronic Engineering and Automation, Shanghai University, Shanghai 200072, China

## Abstract

A symmetric Kullback-Leibler metric based tracking system, capable of tracking moving targets, is presented for a bionic spherical parallel mechanism to minimize a tracking error function to simulate smooth pursuit of human eyes. More specifically, we propose a real-time moving target tracking algorithm which utilizes *spatial histograms* taking into account symmetric Kullback-Leibler metric. In the proposed algorithm, the key spatial histograms are extracted and taken into particle filtering framework. Once the target is identified, an image-based control scheme is implemented to drive bionic spherical parallel mechanism such that the identified target is to be tracked at the center of the captured images. Meanwhile, the robot motion information is fed forward to develop an adaptive smooth tracking controller inspired by the *Vestibuloocular Reflex* mechanism. The proposed tracking system is designed to make the robot track dynamic objects when the robot travels through transmittable terrains, especially bumpy environment. To perform *bumpy-resist capability* under the condition of violent attitude variation when the robot works in the bumpy environment mentioned, experimental results demonstrate the effectiveness and robustness of our bioinspired tracking system using bionic spherical parallel mechanism inspired by head-eye coordination.

## 1. Introduction

Robot vision systems are crucial to recognize and acquire surrounding information for mobile robots. Target tracking, target recognition, surrounding perception, robotic localization, and attitude estimation are the most popular topics in robotics. And the target tracking function has emerged as a significant aspect for* Human Robot Interaction* (*HRI*), camera* Motion-Disturbance Compensation* (MDC), and tracking stabilization.

The robot motion information is commonly used to keep the camera stabilization and compensate small rotation or movements of the camera. These systems used inertial sensors and visual cues to compute the motion information of the camera. Jung and Sukhatme [1] developed a* Kanade-Lucas-Tomasi* (*KLT*) based motion tracking system for a moving target using a single camera on a mobile robot. Hwangbo et al. [2, 3] also developed a gyro-aided KLT feature tracking method that remained robust under fast camera-ego rotation conditions. Park et al. [4] proposed an Extended Kalman Filter (*EKF*) based motion data fusion scheme for visual object tracking by autonomous vehicles. Jia et al. [5] also proposed a scheme of joint of visual features and the vehicle's inertial measurements for visual object identification and tracking. Hol et al. [6] used a multirate EKF by fusing measurements from inertial sensors (accelerometers and rate gyroscopes) and vision to estimate and predict position and orientation (pose) of a camera for robust real-time tracking.

Recently, biomimetic systems were extensively investigated by adopting the movement mechanics of human eye. The development of eyeball's neurophysiology provides a large amount of data and theory foundation for building up the controlling model of eye movement. Among the several types of eye movements, smooth tracking and gaze stabilization play a fundamental role. Lenz et al. [7] developed an adaptive gaze stabilization controller inspired by the Vestibuloocular Reflex (VOR). It integrated inertial and visual information to drive the eyes in the opposite direction to head movement and thereby stabilized the image on the retina under dynamic changes. Shibata's biological oculomotor systems [8] used human-eye's VOR and Optokinetic Reflex (OKR) to improve the gaze stabilization of vision system. A chameleon-inspired binocular “negative correlation” visual system (CIBNCVS) with neck [9] was designed to achieve swift and accurate positioning and tracking. Avni et al. [10] also presented a biologically motivated approach of tracking with independent cameras inspired by chameleon-like visual system. Law et al. [11] described biologically constrained architecture for developmental learning of eye-head gaze control on an iCub robot. Xie et al. [12] proposed a biomimetic control strategy of on-board pan-tilt-zoom camera to stabilize visual tracking from a helicopter based on physiological neural path of eye movement control. Vannucci et al. [13] established an adaptive model for robotic control able to perform visual pursuit with prediction of the target motion. Falotico et al. [14] employed “catch-up” saccade model to fixate the object of interest in case of moving targets in order to obtain a human-like tracking system. Compared with the classical control methods, the advantages of using a bionic controller make the robot easily adapted to transmittable terrains and track moving targets stably. Inspired by the excellent work, we tackle turbulence problem of tracking when the robots travel through bumpy terrains using a tracking system, that is,* bumpy-resist capability*.

Furthermore, with the development of anatomy of human eye, the movement mechanics of the human eye have aroused much interest in bionic engineering. Humanoid robot James [15, 16] was equipped with two artificial eyes, which can pan and tilt independently (totally 4 DOFs.). Thus, the iCub [17, 18] also had two artificial eyes with 3 DOFs, offering viewing and tracking motions. Wang et al. [19] devised a novel humanoid robot eye, which is driven by six pneumatic artificial muscles (PAMs) and rotates with 3 DOFs. Bioinspired actuators and mechanisms have been proposed to pan and tilt a camera with comparable characteristics as a human eye [20, 21]. Tendon-driven robot eye [22] was presented utilizing a mechanical base of the geometry of the eye and of its actuation system behind the implementation of Listing's law. Gu et al. [23] presented an artificial eye implant with shape memory alloys (SMAs) driven by a small servomotor. A miniature artificial compound eye called the curved artificial compound eye (CurvACE) [24] was endowed using similar micromovements to those occurring in the fly's compound eye.

Many bionic eyes have been presented as mentioned above. However, spherical parallel mechanism (SPM) has a compact structure, excellent dynamic performance, and high accuracy; in addition, a 3-DOF SPM is in line with the structural design of the bionic eye. 3-DOF SPMs attract decent amount of interest for this reason. A large number of these 3-DOF SPM bionic eyes have been proposed. An artificial eye [25, 26] for humanoid robots has been devised to be small in size and weight as well as to imitate the high dynamic movements of the human eye. The “Agile Eye” [27] is a high-performance parallel mechanism which has the capability of orienting a camera mounted end effector within a workspace larger than that of a human eye and with velocities and accelerations larger than those of the human eye. Bang et al. [28] design a 3-DOF anthropomorphic oculomotor system to match the human-like eye's performance capabilities. Our mechanism platform is inspired by these excellent works and plays a vital role in tracking dynamic objects.

Tracking a dynamic object when a robot performs its normal motion is common in application. To keep smoothly tracking moving objects, we develop a* bioinspired tracking system that is extensively used when the robot works in bumpy environment or with dynamic disturbance* in this paper. With active robot vision, an image-based feedback tracking system is presented for our bionic SPM to minimize tracking servoing, capable of tracking moving target when the robot moves across in bumpy environment. More specifically, we propose a real-time moving target tracking algorithm which utilizes spatial histograms and symmetric Kullback-Leibler (*SKL*) metric integrated in particle filtering framework to achieve automatic moving target tracking and gaze stabilization. In the proposed algorithm, the key spatial histograms are extracted and taken into particle filtering framework. An image-based feedback control scheme is implemented to drive bionic SPM such that the identified target is to be tracked at the center of the captured images. Meanwhile, the robot motion information is fed forward to develop an adaptive smooth tracking controller bioinspired by the VOR mechanism. To perform good specification, we test our vision stability system under the condition of violent attitude variation when the robot works in* bumpy environment*.

From a robotics point of view, our system is biologically inspired. While smooth tracking is employed to create a consistent perception of the surrounding world, the interaction with environment is also used to adjust the control model involved in the smooth tracking generation. Action and perception are tightly coupled in a bidirectional way: perception triggers an action and the output of action changes the perception. Meanwhile, the robot motion information is fed forward, inspired by the VOR mechanism, to stabilize smooth tracking.

The paper is organized as follows.  Section 2 introduces bionic issues and design of our bionic SPM.  Section 3 proposes visual tracking based on symmetric Kullback-Leibler metric* spatiograms*. Our bionic eye plant control system is described in Section 4. Experimental results are shown in Section 5.  Section 6 presents our conclusion.

## 2. Design of Human-Eye-Inspired PTZ Platform

### 2.1. Human-Eye's Movement Mechanism

**Figure 1:**
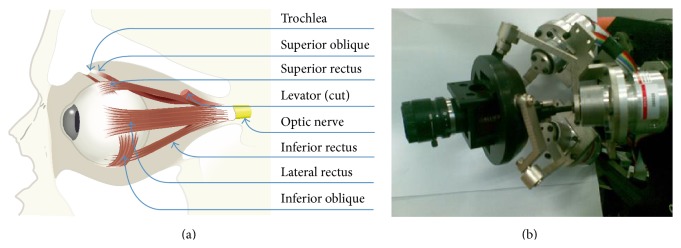
Development of bionic eye plant. (a) Muscles of the eye. Six muscles, arranged in three pairs, control the movements of the eye as shown here in a cutaway view of the eye in its socket or orbit. (b) Structure of our SPM prototype.

Each eye is controlled by three complementary pairs of extraocular muscles, as shown in Figure 1(a). The movement of each eye involves rotating the globe of the eye in the socket of the skull. Because of minimal translation during its movement, the eye can be regarded as a spherical joint with an orientation defined by three axes of rotation (horizontal, vertical, and torsional). But in our implementation and development of a simulator, we view eye movement with no translation for simplicity.

The medial rectus turns eye inward and, thus, lateral rectus outward. Therefore, they form a pair to control the horizontal position of the eye. In contraction to the pair of medial rectus and lateral rectus, the actions of the other two pairs of muscles are more complex. When the eye is centered in the orbit, the primary effect of the superior and inferior rectus is to rotate up or rotate down the eye. However, when the eye is deviated horizontally in the orbit, these muscles also contribute to torsion, the rotation of the eye around the line of sight that determines the orientation of images on the retina.

The primary effect of the superior and inferior obliques is to turn eyes downward and upward when the eye does not deviate from horizontal position. So do superior rectus and inferior rectus. In addition, these muscles also determine the vertical orientation of the eye.

Smooth pursuit eye movements slowly rotate the eyes to compensate for any motion of the visual target and thus act to minimize the blurring of the target's retinal image that would otherwise occur. We implement smooth target tracking, continuously adjusted by visual feedback about the target's image (retinal image).

Kinematic characteristics of SPM and the mechanics of eye movements are very similar [29]. Both have a 3-DOF spherical movement and rotating globe is the center of the sphere. SPM also has a compact structure, excellent dynamic performance, and high accuracy, so 3-DOF SPM is in line with the structural design of the bionic eye to replicate the eye movement.

The eyeball is seen as a sphere, with a rotation center when it rotates. Inspired by the mechanics of eye movements and active robotic vision, we presented a new bionic eye prototype based on SPM, which is made up of an eye-in-hand system as shown in Figure 1(b).

Because the eye is free to rotate in three dimensions, eyeballs can keep retinal images stable in the fovea when they track moving target. In our work, we proposed two main points about structural requirements inspired by the human eyes: (1) camera center must be located at the center of “eyeball” to ensure that the angle of image planes between two different positions keeps identical with the rotation of “eyeball”; (2) in the process of eye movement, any mechanical component except the “eyeball” cannot exceed the plane of the center of the sphere as much as possible to ensure that when the movement of the “eyeball” occurs, they do not block the sight of the camera and do not interfere with the robot face.

### 2.2. Oculomotor Plant Compensation of VOR

In the human-eye VOR, a signal from the vestibular system related to head velocity, which is encoded by semicircular ducts, is used to drive the eyes in the opposite direction to the head movement. The VOR operates in feedforward mode and as such requires calibration to ensure accurate nulling of head movement. The simplicity of this “three-neuron arc,” together with the relatively straightforward mechanics of the eye plant, has long made the VOR an attractive model for experimental and computational neuroscientists seeking to understand cerebellar function. To abolish image motion across the retina, the vestibular signal must be processed by neural circuitry which compensates for the mechanical properties of the oculomotor plant. The VOR is therefore a particular example of motor plant compensation. Horizontal and vertical and angular and linear head movement motivate the appropriate combinations of six extraocular muscles in three dimensions.

### 2.3. Kinematics of Human-Eye-Inspired PTZ Platform

**Figure 2:**
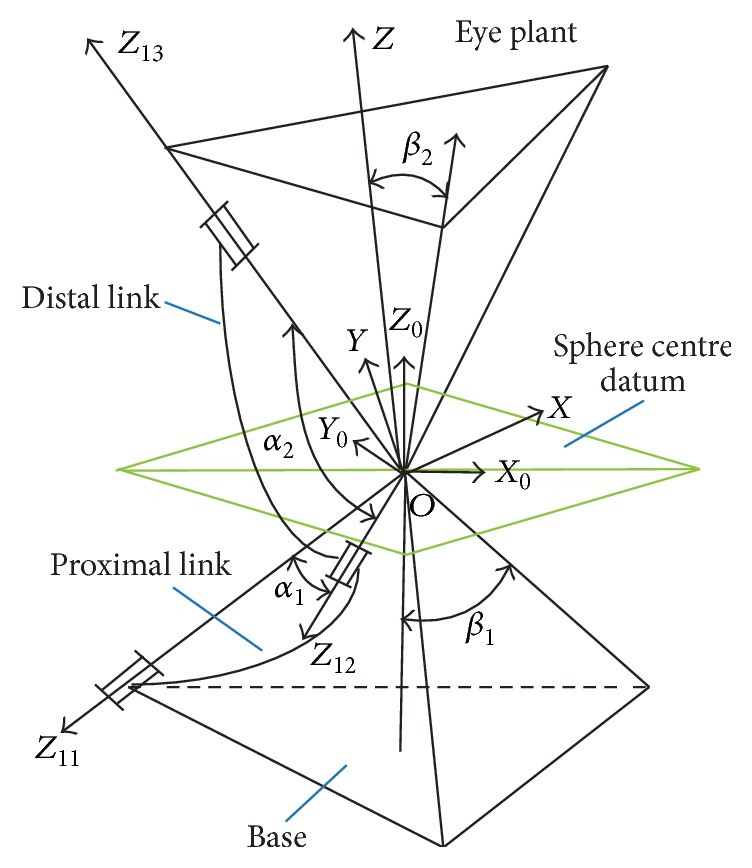
Kinematic sketch of a spherical parallel manipulator.

Spherical parallel mechanism consists of an upper layer and a base, connected by three pairs of identical kinematic subchains as shown in Figure 2. In each chain, there is one fixed revolute joint *z*_*i*_ and two free revolute joints *x*_*i*_ and *y*_*i*_ connecting the proximal link to the distal link and the distal link to the upper layer, respectively. The axes of all revolute joints intersect at a common point *O* which is referred to as the rotational center. The plane passing through the point *O* and becoming parallel with the base is called the sphere center plane, also seen as Listing's plane of eyeball. *α*_1_, *α*_2_, *β*_1_, *β*_2_, and *η* are the parameters of this mechanism, where *α*_1_ and *α*_2_ are the structural angle of the lower link and upper link, *β*_1_ and *β*_2_ are the half-cone angle of the upper platform and the base, and *η* is the structural torsion angle of initial state of the upper platform and the base, namely, the initial torsion angle.

**Figure 3:**
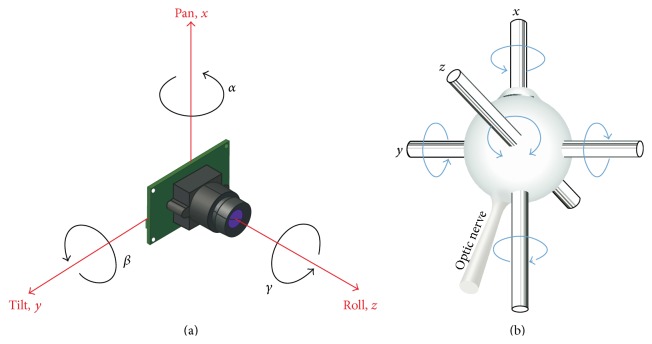
Analogue of camera rotation and eye movement. Our SPM prototype has similar kinematics to human eye.

Figure 2 demonstrates the kinematics of our SPM platform, and the kinematic equation of the SPM is given by [30]

(1) $$\begin{array}{c} \dot{\theta }=J\left(\;\alpha_{1},\alpha_{2},\beta_{1},\beta_{2},\eta ,\phi_{x},\phi_{y},\phi_{z}\;\right)\omega , \end{array}$$

where $\dot{\theta }=\left({\dot{\theta }}_{1},{\dot{\theta }}_{2},{\dot{\theta }}_{3}\right)$ is the angular velocity vector input of the motor, **ω** = (*ω*_*x*_, *ω*_*y*_, *ω*_*z*_) is the angular velocity vector output of the upper platform, and *J* is the Jacobian matrix which is decided by the mechanical parameter (*α*_1_, *α*_2_, *β*_1_, *β*_2_,  and  *η*) and the eyeball posture (*ϕ*_*x*_, *ϕ*_*y*_,  and  *ϕ*_*z*_). *α*_1_ and *α*_2_ are the structural angles of lower link and upper link, respectively. *β*_1_ and *β*_2_ are the angles of base and upper platform. The proposed PTZ platform has similar kinematics to the human eye, as shown in Figure 3.

## 3. SKL-Based Particle Filter Visual Tracking

### 3.1. Spatial Histograms: Spatiogram

Color histogram is one of the common target models which is just a statistic of different colors in the entire picture in proportion without concern for spatial location of each color. Therefore, it is not rather sensitive to rotation but suitable for nonrigid or prone to deformation modeling target objects. Targets based on this model are vulnerable to backgrounds which have similar color distribution or other interference, thereby causing the target tracking failure. In this paper, we improve the particle filter algorithm based on a new target model, spatiogram [31], which adds the pixel coordinate information to the traditional color histogram and uses SKL metric. The second-order spatiogram can be described as follows:

(2) $$\begin{array}{c} h\left(\;b\;\right)=\left\{\;n_{b},\mu_{b},\Sigma_{b}\;\right\},\;b=1,\ldots ,B, \end{array}$$

where *B* is the total number of the intervals and {*n*_*b*_, ***μ***_*b*_, Σ_*b*_} is the probability of each interval, coordinate mean, and covariance matrix, respectively. They can be calculated using the formula as follows:

(3) nb=1N∑j=1Nδjb,μb=1N∗nb∑j=1Nxjδjb,Σb=1N∗nb−1∑j=1Nxj−μbxj−μbTδjb.

*B* is the total number of pixels within the target area, **x**_*j*_ = [*x*_*j*_, *y*_*j*_]^*T*^ is the coordinate position of the *j*th pixel, and *δ*_*jb*_ = 1 denotes that the *j*th pixel is quantized to the *b*th interval, while *δ*_*jb*_ = 0 indicates that the *j*th pixel is quantized to other intervals.

### 3.2. SKL-Based Particle Filter

In order to apply the spatiogram to target tracking, we need to select a method to measure the similarity metrics of the spatial histogram between the targets and the candidate targets. We select the SKL-based coefficient of similarity metrics to measure the similarity of the target spatiogram *h*(*b*) = {*n*_*b*_, ***μ***_*b*_, Σ_*b*_} and candidate target spatiogram *h*′(*b*) = {*n*_*b*_′, ***μ***_*b*_′, Σ_*b*_′}.

Given a spatiogram *h*(*b*) = {*n*_*b*_, ***μ***_*b*_, Σ_*b*_}, we use a Gaussian distribution to describe the spatial distribution of all the pixels in each section. The distribution of the *b*th section can be described as

(4) $$\begin{array}{c} f_{b}\left(\;x\;\right)=\frac{1}{2\pi {\left|\Sigma_{b}\right|}^{1/2}}\exp\left[\;-\frac{1}{2}{\left(\;x-\mu_{b}\;\right)}^{T}\Sigma_{b}^{-1}\left(\;x-\mu_{b}\;\right)\;\right], \end{array}$$

where ***μ***_*b*_ is the mean value of all coordinates of the pixels of the *b*th interval and Σ_*b*_ is the mean covariance matrix of all coordinates of the pixels of the *b*th interval.

The KL distance between the Gaussian distribution *f*_*b*_(**x**) and the Gaussian distribution *f*_*b*_′(**x**) can be obtained by a closed form solution which is calculated using the following formula:

(5) KLfb||fb′=12log⁡Σb′Σb+TrΣb′−1Σb−d+μb−μb′TΣb′−1μb−μb′,

where *d* is the spatial dimension (for spatiogram, *d* = 2). Similarly, we can get the KL distance between the Gaussian distribution *f*_*b*_′(**x**) and the Gaussian distribution *f*_*b*_(**x**):

(6) KLfb′||fb=12log⁡ΣbΣb′+TrΣb−1Σb′−d+μb′−μbTΣb−1μb′−μb.

The SKL distance of the two Gaussian distributions of *f*_*b*_(**x**) and *f*_*b*_′(**x**) is

(7) SKLfb||fb′=12KLfb||fb′+KLfb′||fb,SKLfb||fb′=14TrΣb′−1Σb+TrΣb−1Σb′−2d+14μb−μb′TΣb−1+Σb′−1μb−μb′.

Generally, the ranges of the similarity are [0,1], and the similarity *ψ*_*b*_ of each pair of intervals on the spatiogram can be described as

(8) $$\begin{array}{c} \psi_{b}=\exp\left[\;-SKL\left(\;f_{b},f_{b}^{\prime }\;\right)\;\right]. \end{array}$$

Thus, the similarity of the spatiogram based on SKL distance can be calculated as

(9) ρh,h′∑b=1Bnbnb′ψb=∑b=1Bnbnb′exp⁡−SKLfb,fb′.

According to (11), we can get

(10) ρh,h′=∑b=1Bnbnb·exp⁡−14TrΣb−1Σb′+TrΣb−1Σb′−2d+0=∑b=1Bnbexp⁡−14d+d−2d=∑b=1Bnb=1.

This indicates that the similarity measure of symmetric spatiogram based KL distance ensures that the object has the most similarity to the target.

## 4. Image-Based Feedback and Dynamic Compensation Eye Plant Control

### 4.1. Visual Feedback Scheme

When the target is identified in the image, the visual feedback tracking control strategy is proposed to control the bionic eye plant mechanism to minimize a tracking error function, which is also called eye-in-hand visual servoing [32, 33]. Since the relative distance between the SPM and the moving target is large, if the error function is defined in any 3D reference coordinate frame, coarse estimation of the relative pose between the SPM and the moving target may cause the moving target to fall out of the visual field, while adjusting the SPM servo mechanism, and also affect the accuracy of the pose reached after convergence. In our project, to make tracking control more robust and stable, we define a tracking error function in the visual sensor frame, which is given by [32, 33]

(11) $$\begin{array}{c} e\left(\;t\;\right)=s\left(\;t\;\right)-s^{*}, \end{array}$$

where **s**(*t*) and **s** ^*∗*^ are the measured and desired locations of the centroid of the tracked moving target with respect to the image plane, respectively. In our work, we set **s** ^*∗*^ = [0,0]^*T*^, a constant, which is the centroid of the captured image.

### 4.2. Camera Calibration of Human-Eye-Inspired PTZ Platform

Based on the PTZ visual system, the coordinate system is established as shown in Figure 3. Assume the motion of the object is unknown; how do we control motion of the PTZ platform so that the projection of the moving object is fixed at the center of the image plane, with full consideration of the dynamic effects of the “eyeball”? To make *z*-axis of the camera coordinate system coincide with the target by adjusting the posture of the camera, we have to compensate the offset angle between the camera and the target. We employ a pinhole camera model to obtain a more accurate camera projection. Following the general pinhole camera model, the intrinsic parameter model equation of the camera is given by

(12) $$\begin{array}{c} \left[\begin{array}{c} u\\ v\\ 1 \end{array}\right]=\left[\begin{array}{ccc} f_{x} & 0 & u_{0}\\ 0 & f_{y} & v_{0}\\ 0 & 0 & 1 \end{array}\right]\left[\begin{array}{c} \frac{x_{c}}{z_{c}}\\ \frac{y_{c}}{z_{c}}\\ 1 \end{array}\right], \end{array}$$

where (*u*, *v*) denotes the image coordinate of the target in the image coordinate system. (*u*_0_, *v*_0_) are the coordinates of the principal point. (*x*_*c*_, *y*_*c*_, *z*_*c*_) is the target coordinate in the camera coordinate system. *f*_*x*_ is the scale factor in the *x*-coordinate direction, and *f*_*y*_ is the scale factor in the *y*-coordinate direction.

In order to keep the target tracked in the center of the field, we need to make the target lie on the optical axis. The location of the target which passes through the optical axis is represented by (0,0, *z*_*T*_), where *z*_*T*_ is the distance between the camera and the target. The orientation is

(13) $$\begin{array}{c} \left[\begin{array}{c} x_{c}\\ y_{c}\\ z_{c} \end{array}\right]=\left[\begin{array}{ccc} \cos\alpha & -\sin\alpha & 0\\ \sin\alpha & \cos\alpha & 0\\ 0 & 0 & 1 \end{array}\right]\left[\begin{array}{ccc} \cos\beta & 0 & \sin\beta \\ 0 & 1 & 0\\ -\sin\beta & 0 & \cos\beta \end{array}\right]\left[\begin{array}{ccc} 1 & 0 & 0\\ 0 & \cos\gamma & -\sin\gamma \\ 0 & \sin\gamma & \cos\gamma \end{array}\right]\left[\begin{array}{c} 0\\ 0\\ z_{T} \end{array}\right]. \end{array}$$

Finally, we can deduce the angle offset between the target and camera's line of sight:

(14) α=arctan⁡ufx−u0fx,β=arctan⁡fxfyv−v02fx2+u−u02.

In our implementation, the camera center is located at the center of “eyeball” so that the angle of image planes between two different positions keeps identical with the rotation of “eyeball.” The 3-DOF SPM satisfies the principles of eyeball movement; a camera can be mounted in the SPM and actively oriented (horizontal, vertical, and torsional) around its *x*-axis, *y*-axis, and *z*-axis, respectively. We, considering minimal translation during eye's movement, implement our eye plant system with no translation for simplicity. So our visual tracking strategy is applicable to all SPMs with no translation.

**Figure 4:**
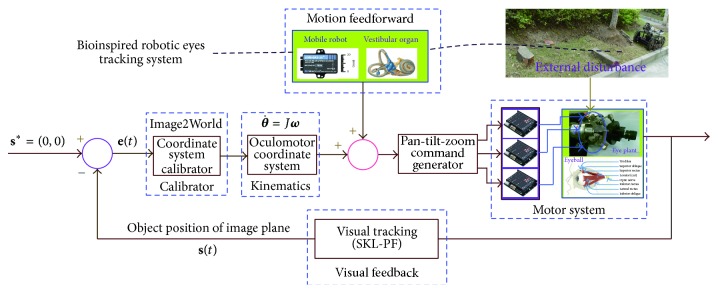
Tracking control scheme. Camera means human's eyeball; IMU means canals.

In the visual tracking section, we give how to determine the position of the moving object. The relative position determines our visual tracking strategy. Eye rotation about the vertical “*z*-axis” is controlled by the lateral and medial rectus muscles, which results in eye movements to left or right. Rotation about the transverse “*y*-axis” is controlled by the superior and inferior rectus muscles, which elevates and depresses the eye. Finally, rotations about the anteroposterior “*x*-axis” result in counterclockwise as well as upward and downward eye motion. See Figures 1(a) and 1(b). Our model receives visually guided signal to control eye plant; see (14). Meanwhile, the robot motion information is fed forward into control loop. Our whole bioinspired tracking system is illustrated in Figure 4. It is known that the VOR is basically driven by the signals from vestibular apparatus in the inner ear. The semicircular canals (SCs) detect the head rotation and drive the rotational VOR; on the other hand, the otoliths detect the head translation and drive the translational VOR. Anatomists and physiologists tend to engage in the VOR as a simple neural system mediated by a three-neuron arc and displaying a distinct function. Starting in the vestibular system, SCs get activated by head rotation and send their impulses via the vestibular nerve through brainstem neurons and end in oculomotor plant. Here, we use IMU to acquire pose change of eye from the robot.

When the robot works in the bumpy environment, rigid bumps and pulse jitter cause the occurrence of significant turbulence with high frequency and posture change with lower frequency. Therefore, the motion information of the robot is acquired and fed forward into the controller to compensate the external disturbance. In [34], an active compensation model of visual error is proposed according to the principle of VOR in (15). Here, we use our proposed bioinspired controller to compensate motion disturbance caused by bumpy jitter. Hence,

(15) Es=Hs−αTvTns2sTv+1sTn+1+esλs+γ+ke−lssTqTnsTn+1,

where *e*(*s*) = −(*H*(*s*) + *E*(*s*)) is slide error of retina, *H*(*s*) denotes the rotation angle of head, and *E*(*s*) means the rotation angle of eyeball. *α*, *λ*, and *γ* represent the gains of the velocity signal of head rotation, the velocity signal of retina slide, and the spike of nerve fibers caused by the displayment of retina, respectively. *k* is the compensation weight value of flocculus caused by error signal of retina. In our system, *α*, *λ*, and *γ* are equal to 1 and *k* = 2.5. Combining position compensation with speed compensation of eyeball, our system is used to build a smooth tracking system.

## 5. Experiments and Results

**Figure 5:**
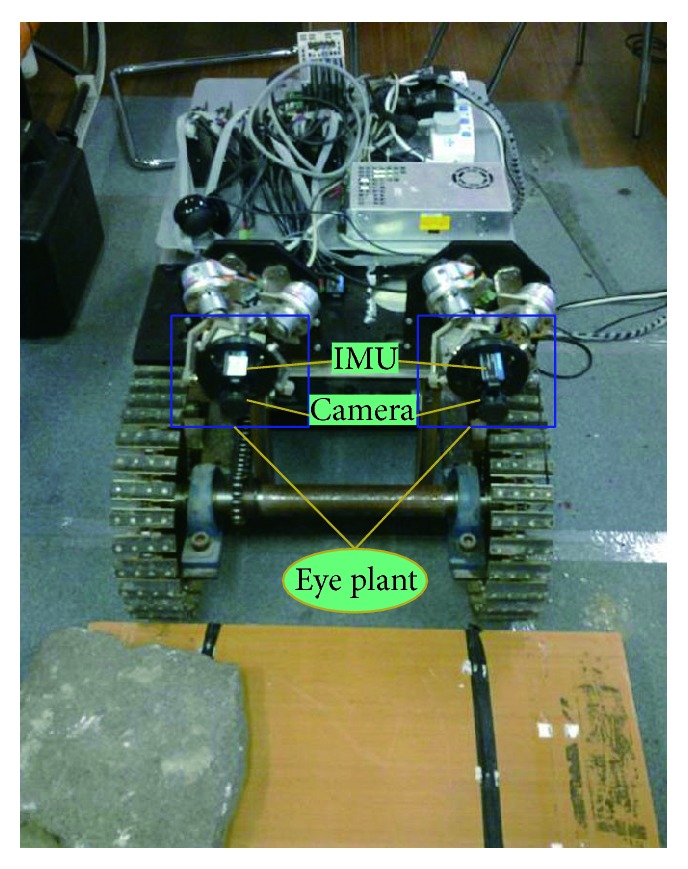
Experimental platform based on Tracked Mobile Robot.

To prove that the proposed image-based feedback tracking system based on our developed eye-in-hand prototype is able to orient a camera with the required orientation changes, especially its dynamic disturbance resistance capability and SPM-based structural dexterity, closed-loop control experiments were performed. We design an experimental platform based on a tracked robot, as shown in Figure 5. A variety of obstacles are placed on the tracked robot's path to simulate a real harsh environment. We introduced the used joint space control architecture in [35]. In the chosen control approach, the desired camera orientation is transformed to linear actuator set points using the inverse kinematics. Thus, here only a brief overview of the architecture and exemplary control results are presented.

To measure angular velocities of “eyeball” in three axes, we employ the attitude sensor 3DM-GX-25TM. The device offers a range of output data quantities from fully calibrated inertial measurements (acceleration, angular rate, and deltaAngle and deltaVelocity vectors) to computed orientation estimates, including pitch, roll, and heading (yaw) or rotation matrix. All quantities are fully temperature compensated and are mathematically aligned to an orthogonal coordinate system.

In addition, the image information is gained by using a high-speed camera (Guppy F-033C), which is connected to the IEEE 1394 card installed in a PC with Intel Core CPU which acquires the video signal. The camera is an ultracompact, inexpensive VGA machine vision camera with CCD sensor (Sony ICX424). At full resolution, it runs up to 58 fps. We employ a pinhole camera model to obtain a more accurate camera projection. Camera calibration was repeated ten times to seek an approximate camera calibration matrix $K=\left[\begin{array}{ccccccccc} f_{x} & 0 & 0; & 0 & f_{y} & 0; & u_{0} & v_{0} & 1 \end{array}\right]$. A camera is calibrated using chessboard [36]. Here, we employ a pinhole camera model to obtain a more accurate camera projection. Following the general pinhole camera model, the parameters contained in *K* are called the internal camera parameters or the internal orientation of the camera. See [37] for more details.

Figure 4 shows our tracking control scheme. We implemented smooth target tracking (smooth pursuit) to ensure the target located in the field of view, continuously adjusted by visual feedback about the target's image. Image is captured from camera (retinal image) and IMU measures the robot body's movement to compensate dynamic disturbance.

Supposing that we do not know the motion of the tracked object, how do we control the motion of the “eyeball” to ensure that the moving object is fixed at the centroid of the image plane?

**Figure 6:**
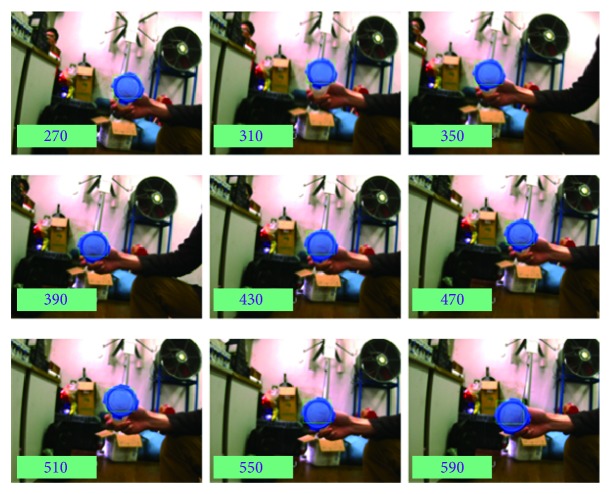
Moving object tracked in the laboratory.

**Figure 7:**
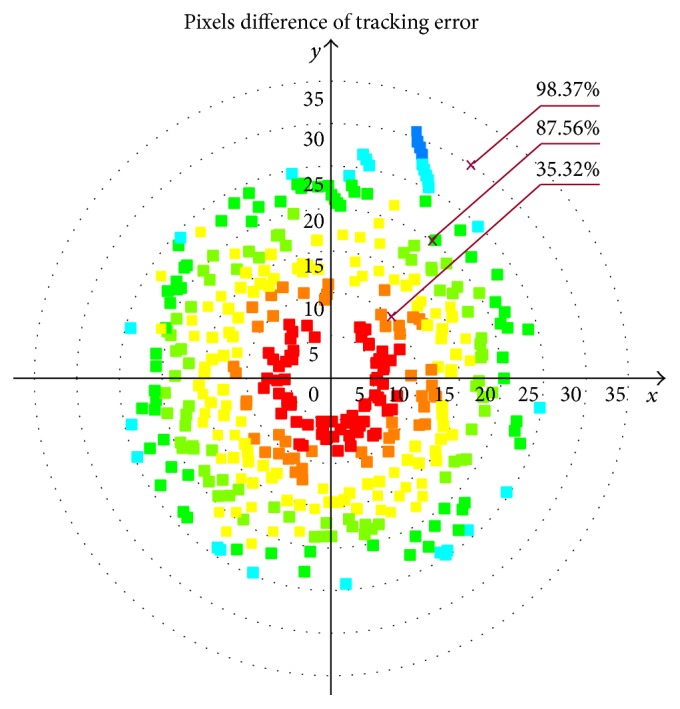
Pixel difference of *x* and *y* directions.

In the process of tracking moving target, the tracking algorithm should be robust to appearance variations introduced by occlusion, illumination changes, and pose variations. In our library environment, the proposed algorithm can relocate the target when object appearance changes due to illumination, scale, and pose variations. Once the moving target is located, the “eyeball” should keep images stable in the field of view (center of image). That is, target position fluctuates at zero. See Figures 6 and 7.  Figure 6 gives some snapshots of tracking results and demonstrates that the moving target is located in the field of view. Meanwhile, extensive experiments are conducted to perform bumpy-resist capability.  Figure 7 illustrates the pixel difference in *x* and *y* direction. Smaller eyeball errors accompanying larger postural changes can be good proofs of good bumpy-resist capability and VOR function.

**Figure 8:**
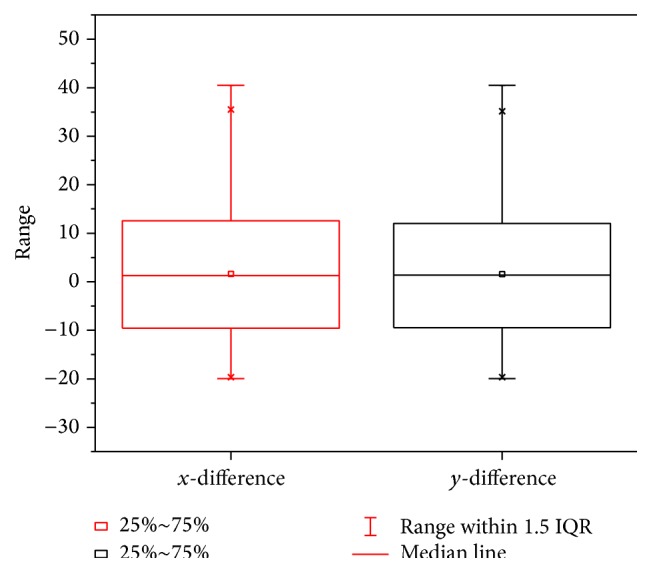
Statistics of *x* and *y* direction difference.

Figures 7 and 8 show the performance of the proposed tracking system on the tracked robot running across bumpy environment. The result of statistics shows that 98.37% of tracking errors, including *x* and *y* direction difference, have fallen into the range of <30 pixels, as shown in Figure 7. The statistics of *x* and *y* direction pixel difference are demonstrated in Figure 8. In our test, as the tracked robot platform travels through the rough ground full of obstacles, rigid bumps and pulse jitter cause the occurrence of significant turbulence with high frequency and the oscillatory posture changes with lower frequency, which makes tracking effect slightly larger than the data recorded in the literature, such as [13, 14]. But our experiments are established under relatively harsh environmental conditions, and the effect achieved is objective. Tracking effects still stay in a controllable range like the above situation. Apparently, this indicates that the system has robustness.

**Figure 9:**
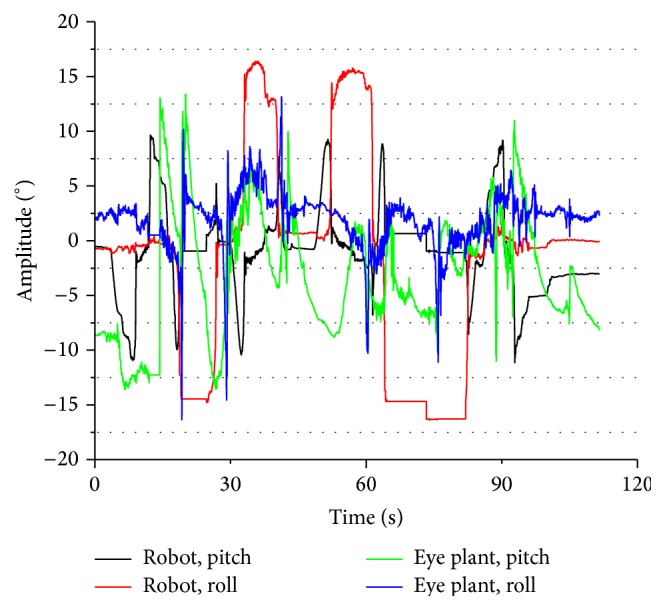
Experimental results of robot's and eye plant's pose.

**Figure 10:**
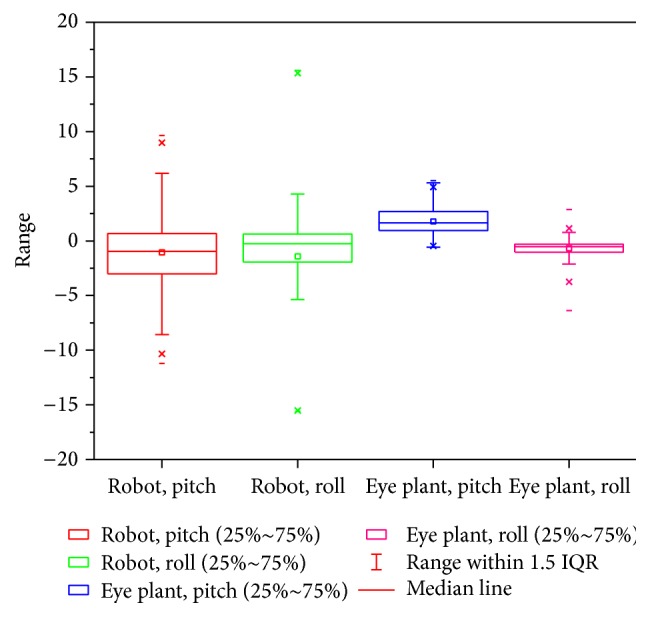
Statistics of robot's and eye plant's pose.

In our actual situation, we install three IMUs on the tracked robot and eye plants to measure the pose changes. We recorded the angle variances to validate the system bumpy-resist capability that the eyeball moves on the opposite direction according to position compensation and velocity compensation when the tracked robot's pose changes. In other words, the robot pose variance information is fed forward into controller to form a head-eye coordination system. Figures 9 and 10 show the experimental results of tracked robot's and eye plant's pose changes on the tracked robot in bumpy environment. In addition, the large tracking errors happen when the robot encounters instantaneous postural changes. Nonetheless, quick returns to lower errors of eyeball verify good robustness of the bionic visual tracking system and high dexterity of the SPM-based bionic eye. Obviously, these variances reflect good stability of the tracking system.

## 6. Conclusion

To accurately replicate the human vision system, we presented a 3-DOF “eyeball” in the directions of horizontal, vertical, and torsional axes according to the mechanics of eye movements. Thus, an image-based visual feedback tracking system is presented to minimize a tracking error function, capable of tracking moving target. More specifically, the proposed real-time moving target tracking algorithm utilizes spatial histograms and symmetric Kullback-Leibler metric integrated into particle filtering framework to achieve automatic moving target identification and gaze stabilization. Meanwhile, the robot motion information is fed forward to develop an adaptive smooth tracking controller bioinspired by the VOR mechanism. The experimental results demonstrate that our algorithm is effective and robust in dealing with moving object tracking and can always keep the target at the center of the camera to avoid tracking failure. Furthermore, as the tracked robot platform travels through the rough ground full of obstacles, rigid bumps and pulse jitter cause the occurrence of significant turbulence with high frequency and the oscillatory posture changes with lower frequency. Tracking effects still stay in a controllable range and this indicates that the system has bumpy-resist capability.

## References

[1] Jung B., Sukhatme G. S. (2010). Real-time motion tracking from a mobile robot. *International Journal of Social Robotics*.

[2] Hwangbo M., Kim J.-S., Kanade T. (2011). Gyro-aided feature tracking for a moving camera: fusion, auto-calibration and GPU implementation. *The International Journal of Robotics Research*.

[3] Hwangbo M., Kim J.-S., Kanade T. Inertial-aided KLT feature tracking for a moving camera.

[4] Park J., Hwang W., Kwon H., Kim K., Cho D.-I. D. (2013). A novel line of sight control system for a robot vision tracking system, using vision feedback and motion-disturbance feedforward compensation. *Robotica*.

[5] Jia Z., Balasuriya A., Challa S. (2008). Sensor fusion-based visual target tracking for autonomous vehicles with the out-of-sequence measurements solution. *Robotics and Autonomous Systems*.

[6] Hol J. D., Schön T. B., Luinge H., Slycke P. J., Gustafsson F. (2007). Robust real-time tracking by fusing measurements from inertial and vision sensors. *Journal of Real-Time Image Processing*.

[7] Lenz A., Balakrishnan T., Pipe A. G., Melhuish C. (2008). An adaptive gaze stabilization controller inspired by the vestibulo-ocular reflex. *Bioinspiration and Biomimetics*.

[8] Shibata T., Schaal S. (2001). Biomimetic gaze stabilization based on feedback-error-learning with nonparametric regression networks. *Neural Networks*.

[9] Xu H., Xu Y., Fu H., Xu Y., Gao X. Z., Alipour K. (2014). Coordinated movement of biomimetic dual PTZ visual system and wheeled mobile robot. *Industrial Robot*.

[10] Avni O., Borrelli F., Katzir G., Rivlin E., Rotstein H. (2008). Scanning and tracking with independent cameras-a biologically motivated approach based on model predictive control. *Autonomous Robots*.

[11] Law J., Shaw P., Lee M. (2013). A biologically constrained architecture for developmental learning of eye–head gaze control on a humanoid robot. *Autonomous Robots*.

[12] Xie S., Luo J., Gong Z., Ding W., Zou H., Fu X. Biomimetic control of pan-tilt-zoom camera for visual tracking based-on an autonomous helicopter.

[13] Vannucci L., Cauli N., Falotico E., Bernardino A., Laschi C. Adaptive visual pursuit involving eye-head coordination and prediction of the target motion.

[14] Falotico E., Zambrano D., Muscolo G. G., Marazzato L., Dario P., Laschi C. Implementation of a bio-inspired visual tracking model on the iCub robot.

[15] Jamone L., Fumagalli M., Metta G., Natale L., Nori F., Sandini G. Machine-learning based control of a human-like tendon-driven neck.

[16] Nori F., Jamone L., Sandini G., Metta G. Accurate control of a human-like tendon-driven neck.

[17] Tsagarakis N. G., Metta G., Sandini G. (2007). iCub: the design and realization of an open humanoid platform for cognitive and neuroscience research. *Advanced Robotics*.

[18] Leitner J., Harding S., Frank M., Förster A., Schmidhuber J. (2013). An integrated, modular framework for computer vision and cognitive robotics research (icVision). *Biologically Inspired Cognitive Architectures*.

[19] Wang X.-Y., Zhang Y., Fu X.-J., Xiang G.-S. (2008). Design and kinematic analysis of a novel humanoid robot eye using pneumatic artificial muscles. *Journal of Bionic Engineering*.

[20] Lee Y.-C., Lan C.-C., Chu C.-Y., Lai C.-M., Chen Y.-J. (2013). A pan–tilt orienting mechanism with parallel axes of flexural actuation. *IEEE/ASME Transactions on Mechatronics*.

[21] Lan C.-C., Lee Y.-C., Jiang J.-F., Chen Y.-J., Wei H.-Y. Design of a compact camera-orienting mechanism with flexural pan and tilt axes.

[22] Cannata G., Maggiali M. (2008). Models for the design of bioinspired robot eyes. *IEEE Transactions on Robotics*.

[23] Gu J., Meng M., Cook A., Faulkner M. G. (2000). A study on natural movement of artificial eye implant. *Robotics and Autonomous Systems*.

[24] Colonnier F., Manecy A., Juston R. (2015). A small-scale hyperacute compound eye featuring active eye tremor: application to visual stabilization, target tracking, and short-range odometry. *Bioinspiration & Biomimetics*.

[25] Villgrattner T., Schneider E., Andersch P., Ulbrich H. (2011). Compact high dynamic 3 DoF camera orientation system: development and control. *Journal of System Design and Dynamics*.

[26] Villgrattner T., Ulbrich H. Optimization and dynamic simulation of a parallel three degree-of-freedom camera orientation system.

[27] Gosselin C. M., St. Pierre E., Gagné M. (1996). On the development of the agile eye. *IEEE Robotics & Automation Magazine*.

[28] Bang Y.-B., Paik J. K., Shin B.-H., Lee C. (2006). A three-degree-of-freedom anthropomorphic oculomotor simulator. *International Journal of Control, Automation and Systems*.

[29] Refaat S., Hervé J. M., Nahavandi S., Trinh H. (2007). Two-mode overconstrained three-DOFs rotational-translational linear-motor-based parallel-kinematics mechanism for machine tool applications. *Robotica*.

[30] Li C., Xie S., Li H., Wang D., Luo J. (2010). Design of bionic eye based on spherical parallel mechanism with optimized parameters. *Robot*.

[31] Birchfield S. T., Rangarajan S. Spatiograms versus histograms for region-based tracking.

[32] Chaumette F., Hutchinson S. (2006). Visual servo control. I. Basic approaches. *IEEE Robotics and Automation Magazine*.

[33] Chaumette F., Hutchinson S. (2007). Visual servo control. II. Advanced approaches [Tutorial]. *IEEE Robotics and Automation Magazine*.

[34] Li H., Luo J., Li C., Li L., Xie S. (2011). Active compensation method of robot visual error based on vestibulo-ocular reflex. *Jiqiren/Robot*.

[35] Li C., Xie S., Li H., Miao J., Xu Y., Luo J. (2011). System design and study on bionic eye of spherical parallel mechanism based on attitude closed-loop control. *Jiqiren/Robot*.

[36] Zhang Z. (2000). A flexible new technique for camera calibration. *IEEE Transactions on Pattern Analysis and Machine Intelligence*.

[37] Hartley R., Zisserman A. (2003). *Multiple View Geometry in Computer Vision*.

